# The Role of Palliative Care in New Perioperative DNR Orders: Risk Factors and Timing

**DOI:** 10.1093/geroni/igaf122.3379

**Published:** 2025-12-31

**Authors:** Sarah Remer, Marcia Russell, Ronnie Rosenthal, Clifford Ko

**Affiliations:** The American College of Surgeons, Chicago, Illinois, United States; David Geffen School of Medicine at UCLA, Los Angeles, California, United States; Yale School of Medicine, New Haven, Connecticut, United States; David Geffen School of Medicine at UCLA, Los Angeles, California, United States

## Abstract

As the population ages, there is an increased need for comprehensive goals of care (GOC) discussions which often include Do-Not-Resuscitate (DNR) orders. DNR orders are being increasingly studied, but it remains unknown how palliative care consultation (PCC) relates in older adult surgical patients. The American College of Surgeons National Surgical Quality Improvement Program Geriatric Surgery Verification pilot program data was utilized from January 1, 2015 to December 31, 2019. The primary outcome was new DNR order within 30 days of primary operation for patients 65 and older. The association between PCC and DNR was assessed including timing. There were 44,172 patients, 2.7% of which had a new DNR order. 21% were placed preoperatively, 33% the day of surgery, 46% postoperatively. Of the patients who had DNR orders placed, 32% had PCCs versus 0.5% of those who did not have DNR orders (p < 0.0001). 45% of patients with both new DNR and PCC had them placed on the same day, 10% of PCC were before new DNR, and 45% were after. 57% of PCCs were placed ≤1 day prior to death and 49% of PCCs were placed >7 and ≤30 days postoperatively. After adjustment, PCC was significantly associated with new DNR (OR 9.95; 95% CI 6.42-15.42). Almost half of PCC and new DNR orders occur on the same day. Half of PCCs are on the same day as death signifying need for earlier GOC discussions. Further research is needed to better identify risk factors for earlier PCC to inform GOC.

